# Calculation of Haem Iron Intake and Its Role in the Development of Iron Deficiency in Young Women from the Australian Longitudinal Study on Women’s Health

**DOI:** 10.3390/nu9050515

**Published:** 2017-05-19

**Authors:** Angela J. Reeves, Mark A. McEvoy, Lesley K. MacDonald-Wicks, Daniel Barker, John Attia, Allison M. Hodge, Amanda J. Patterson

**Affiliations:** 1 Nutrition and Dietetics, School of Health Sciences, Faculty of Health and Medicine, University of Newcastle, Callaghan, Newcastle, NSW 2308, Australia; angela.reeves@uon.edu.au (A.J.R.); lesley.wicks@newcastle.edu.au (L.K.M.-W.); 2 Centre for Clinical Epidemiology & Biostatistics, Hunter Medical Research Institute, School of Medicine & Public Health, University of Newcastle, Callaghan, Newcastle, NSW 2308, Australia; mark.mcevoy@newcastle.edu.au (M.A.M.); daniel.barker@newcastle.edu.au (D.B.); john.attia@newcastle.edu.au (J.A.); 3 Cancer Epidemiology Centre, Cancer Council Victoria, Centre for Epidemiology and Biostatistics, Melbourne School of Population and Global Health, The University of Melbourne, 207 Bouverie Street, Melbourne, VIC 3010, Australia; allison.hodge@cancervic.org.au

**Keywords:** haem iron, iron deficiency, longitudinal analysis, women’s health

## Abstract

Total iron intake is not strongly associated with iron stores, but haem iron intake may be more predictive. Haem iron is not available in most nutrient databases, so experimentally determined haem contents were applied to an Australian Food Frequency Questionnaire (FFQ) to estimate haem iron intake in a representative sample of young women (25–30 years). The association between dietary haem iron intakes and incident self-reported diagnosed iron deficiency over six years of follow-up was examined. Haem iron contents for Australian red meats, fish, and poultry were applied to haem-containing foods in the Dietary Questionnaire for Epidemiological Studies V2 (DQESv2) FFQ. Haem iron intakes were calculated for 9076 women from the Australian Longitudinal Study on Women’s Health (ALSWH) using the DQESv2 dietary data from 2003. Logistic regression was used to examine the association between haem iron intake (2003) and the incidence of iron deficiency in 2006 and 2009. Multiple logistic regression showed baseline haem iron intake was a statistically significant predictor of iron deficiency in 2006 (Odds Ratio (OR): 0.91; 95% Confidence Interval (CI): 0.84–0.99; *p*-value: 0.020) and 2009 (OR: 0.89; 95% CI: 0.82–0.99; *p*-value: 0.007). Using the energy-adjusted haem intake made little difference to the associations. Higher haem iron intake is associated with reduced odds of iron deficiency developing in young adult Australian women.

## 1. Introduction

Iron deficiency is recognised as the most prevalent nutrient deficiency globally [1]. Women of childbearing age are at particularly high risk of iron deficiency due to additional iron losses from menstrual bleeding and increased iron requirements during pregnancy [1]. In 2000, the AusDiab study reported the prevalence of iron deficiency amongst Australian women aged 25–49 years as 20.3% [2]. Iron deficiency has negative health effects, impairing physical, immune, and cognitive functioning [3]. Further research is therefore warranted to identify dietary predictors of iron deficiency in premenopausal adult women. 

Haem iron, derived from animal flesh foods, namely red meat, fish, and poultry, exists in the reduced state (Fe^2+^) and this facilitates greater absorption compared to oxidised non-haem iron (Fe^3+^). Haem iron thus has greater bioavailability [4,5,6]. Between 15–25% of consumed haem iron is absorbed compared with 5–12% for non-haem iron [4,7,8]. Hunt (2003) showed that women aged 20–44 years with low iron stores could increase total iron (haem and non-haem iron) absorption by consuming a highly bioavailable diet [9]. The high-bioavailability diet contained 294 g of meat (red or white) and ≥75 mg of ascorbic acid as an absorption enhancing factor, at every main meal. Daily phytate intake in foods such as legumes or wholegrain cereals/breads was also limited as this reduces non-haem iron absorption. In contrast, the low-bioavailability diet only contained limited amounts of white meat, and legumes or wholegrain cereals/breads were consumed at every meal. Non-haem iron absorption, measured by isotopic labelling of the two day menu, from the high-bioavailability diet was reported as 11.1% compared with 2.3% for the low-bioavailability diet. Haem iron absorption increased from 33% for the low-bioavailability diet to 40% in the high-bioavailability diet [9]. 

While total iron absorption is increased by highly bioavailable diets, there is no strong evidence that total iron intake directly influences iron stores (deficient or replete) [10,11]. Limited evidence suggests haem iron intake may be predictive of iron stores [12,13]. Two separate experimental trials with young adult women have successfully shown that dietary manipulation to increase haem iron intake can improve iron stores [14,15]. 

Haem iron values are not readily available in food composition databases and as such, most dietary assessment tools cannot be used to estimate haem iron intakes. For this reason, several models were previously developed to estimate the haem iron contents of meats, most notably, the Monsen et al. model created in 1978. In this model, haem iron in meat was attributed the value of 40% of the total iron [16]. However an experimental study performed by Rangan et al. in 1997 showed that the Monsen et al. model underestimated the haem iron values of Australian meats [17]. Thus, there is a need to describe the haem iron intake of Australian populations using reliable and valid methods in order to understand the role of haem iron in the development of iron deficiency in high-risk groups, such as young adult women. 

There were two distinct aims of this study. The first was to estimate haem iron intake in a representative sample of young Australian women aged 25–30 years participating in the Australian Longitudinal Study of Women’s Health (ALSWH). The second aim was to examine longitudinally the association between haem iron intake in 2003 and the incidence of self-reported diagnosed iron deficiency three and six years later. It was hypothesised that a low haem iron intake would be predictive of self-reported diagnosed iron deficiency. 

## 2. Subjects and Methods

### 2.1. Subjects 

The Australian Longitudinal Study on Women’s Health (ALSWH) is a prospective cohort study that commenced in 1996. A representative sample of 40,394 Australian women were recruited through the Medicare database, which includes all permanent Australian residents. Women were randomly sampled from three age cohorts—women born from 1973–1978 (young cohort *n* = 14,247), women born from 1946–1951 (mid-age cohort *n* = 13,715), and women born from 1921–1926 (older cohort *n*= 12,432). There is an over-representation of women from rural and remote areas of Australia in each age cohort, as these women were sampled at twice the rate of women living in urban areas. Self-reported health data is collected from participants via mailed written surveys. Since 1996 each cohort has been surveyed every three years. Ethics approval for the ALSWH was granted by the ethics committees from the University of Newcastle (approval code H-076-0795) and the University of Queensland (approval code 2001000224) [18]. Further details of the cohort profiles have been reported elsewhere [19].

In the present study, haem iron intake for women in the young cohort was calculated at survey three in 2003 and then modelled against self-reported diagnosed iron deficiency at survey four in 2006 and survey five in 2009. In 2003, participants were aged 25–30 years. Response rates in 2003, 2006, and 2009 were 65%, 67%, and 61%, respectively. Death, frailty, withdrawal from the study, failure to return surveys, or the inability to contact participants accounted for loss to follow-up [18]. Women who completed survey five were not meaningfully different to the non-responders regarding age, marital status, or area of residence at baseline. This suggests that this cohort maintained good generalizability over a long period of time [20]. Those who reported iron deficiency in 2003 (*n* = 1386) were removed from the sample in order to examine the association between baseline dietary haem iron intake and incident cases of self-reported diagnosed iron deficiency. Participants with 0 mg/day intake of haem iron in 2003 (*n* = 55) were assumed to be vegetarian and were also excluded as the aim of this investigation was to determine the influence of haem iron intake on the development of self-reported diagnosed iron deficiency in non-vegetarians.

#### 2.1.1. Dietary Questionnaire for Epidemiological Studies 

Dietary data were collected using a validated 74 item Food Frequency Questionnaire (FFQ) known as the Dietary Questionnaire for Epidemiological Studies (DQESv2) [21]. The DQESv2 was developed by Cancer Council Victoria (CCV) and validated in Australian women of childbearing age against 7-day food diaries [22]. The DQESv2 asked participants to indicate the portion, frequency, and types of foods consumed over the previous 12 months. For the present study, DQESv2 responses were analysed with CCV software using the Australian Food Composition database NUTTAB 95. The output included both nutrient intake data and the amount of each food item consumed by participants in grams per day. 

#### 2.1.2. Exposure Variable: Calculating Haem Iron 

Whilst total iron intake was provided from the DQESv2 analysis, haem iron values are not available in the Australian Food Composition database and therefore haem iron values had to be generated. Fifteen food items were identified in the DQESv2 that contained haem iron, including twelve flesh foods (beef, veal, chicken, lamb, pork, bacon, ham, salami, sausages, grilled or steamed or baked fish, fried fish, and canned fish) and three mixed food items (meat pie, pizza, and hamburger). The haem iron content of these food items was calculated based on the results of a study by Rangan et al., who used the modified Hornsey method [17]. 

Flesh foods from the DQESv2 were assigned the mean cooked wet weight percentage of haem iron contained in a matching flesh food product experimentally analysed by Rangan et al. When a combination of flesh foods analysed by Rangan et al. was needed to adequately represent one DQESv2 flesh food, the percentages of haem iron in the corresponding meats from Rangan et al. were averaged (see Table 1 footnote). The percentage of haem iron, expressed as a decimal figure to two places, was multiplied by the amount of total iron in the flesh food, taken from the DQESv2 database, to calculate the haem iron value (mg/100 g) (Table 1). Daily haem iron intakes were individually calculated for participants based on the amount of foods containing haem iron they were consuming each day.

#### 2.1.3. Outcome Variable: Self-Reported Diagnosed Iron Deficiency

The primary outcome for this study was self-reported diagnosed iron deficiency, which was ascertained using an identical question relating to the diagnosis of iron deficiency in survey three (2003), survey four (2006), and survey five (2009). Participants were asked: “In the last three years, have you been diagnosed or treated for low iron (iron deficiency or anaemia)?” [23,24,25]. 

#### 2.1.4. Covariate Selection 

**Figure 1:**
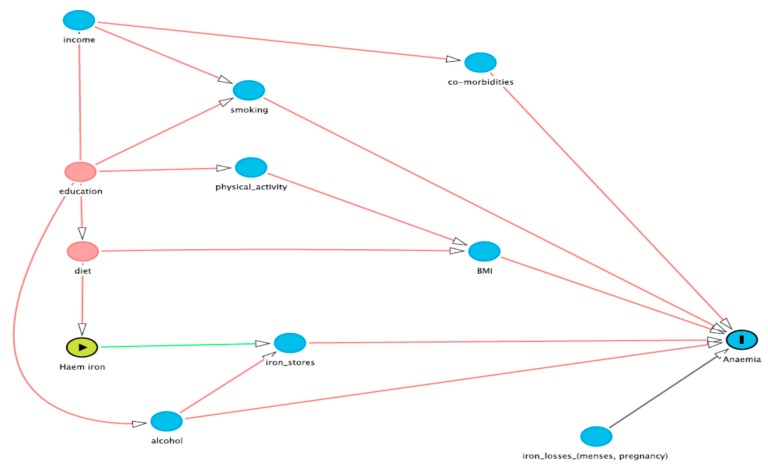
Directed Acyclic Graph (DAG) for the identification of potential confounders. The exposure of interest is haem iron and the outcome of interest is self-reported diagnosed anaemia. The directed acyclic graph makes explicit the causative model we are testing and the relationship of the co-variates. In order to identify confounders, the arrows coming out of haem iron are removed, since these are causative, and any “back door” paths that remain between haem iron and anaemia are identified. The back door paths need not follow the direction of the arrows. Adjusting for a co-variate “closes” the back door path, i.e., removes confounding, assuming that the co-variate is measured without error. A back door path is considered already closed if it passes through a “collider”, i.e., a node with 2 arrows pointing into it. Adjusting for a collider re-opens that path and increases the potential confounding.

A Directed Acyclic Graph (DAG) [26] was used to explore the associations between the exposure and outcome variables, after the literature revealed several factors for consideration as potential confounding variables. From the causal diagram shown in Figure 1, four potential confounders were identified, namely income, education, smoking status, and alcohol consumption. This choice was also confirmed by examining the statistically significant associations of each potential confounder with both haem iron intake and iron deficiency, in accordance with the definition of a confounding variable [27]. Income was divided into three categories: individual income < $37,000, individual income > $37,000, and those who did not know/did not want to report their income (not reported). Education was divided into three groups: no formal qualifications; Year 10 or a trade/certificate; Year 12 and University or Higher Degree. Smoking status was analysed as a continuous variable, defined as the number of cigarettes smoked weekly. Alcohol consumption was also included as a continuous variable, defined as the number of standard drinks consumed weekly. Risk factors for iron deficiency such as recent childbirth (yes or no) or heavy menstrual blood loss (sometimes vs. often) were not treated as confounding variables in the analysis as these variables were not associated with both haem iron intake and iron deficiency [27].

### 2.2. Statistical Analysis

Statistical analysis was conducted using the STATA IC software version 12 (StataCorp LP, College Station, TX, USA). Participant characteristics measured in survey three at baseline in 2003 were tested for association with iron deficiency at survey four in 2006 and survey five in 2009 separately. The means of normally distributed continuous variables were compared in those with or without iron deficiency using two sample *t*-tests. The Chi-squared test was used to compare categorical variables. Non-parametric rank sum analyses, with reported median and interquartile range (IQR), were used to explore skewed continuous distributions.

Logistic regression models investigated baseline haem iron intake as a predictor of iron deficiency at three years (survey four) and six years (survey five) follow-up. In the first step, univariate logistic regression was used to examine the association for surveys four and five. A significance level of 0.05 was applied. The odds ratios, 95% confidence intervals, and *p*-values were reported. A second step used multivariate logistic regression to examine the association between baseline haem iron intake and iron deficiency at surveys four and five with adjustments for income, education, smoking status, and alcohol consumption. The final step repeated this analysis, but haem iron intake was adjusted for total energy intake using Willett’s residual method [28]. Each logistic regression model was assessed for suitability using the Hosmer and Lemeshow goodness-of-fit test. 

## 3. Results

The prevalence of iron deficiency at baseline in 8942 women was 15.5% (*n* = 1386). After removing the existing cases, the incidence of iron deficiency in the 6147 women remaining at survey four (2006) and 5365 women remaining at survey five (2009) was 12.9% (*n* = 794) and 14.3% (*n* = 766), respectively. Iron intakes from flesh foods and mixed foods for the study sample at survey three are shown in Table 1. Haem iron intakes for the study sample ranged from 0.003 mg/day to 35.3 mg/day, with a median haem iron intake of 1.39 mg/day (IQR = 1.23).

Beef and chicken were consumed by the study sample in the greatest quantities and thus contributed the greatest amount of dietary haem iron daily (0.48 mg/day and 0.18 mg/day, respectively). Lamb was the next greatest contributor to haem iron intake (0.13 mg/day), though daily consumption (g) was significantly lower (9 g) than for beef (28 g) and chicken (26 g). 

Baseline characteristics of the study sample and associations with iron deficiency at survey five are described in Table 2. Women with iron deficiency at survey five were more likely to be earning less than $37,000 annually and to have completed tertiary education, although this was not a significant difference. However, they were consuming significantly less haem iron daily than those not diagnosed, and were more likely to have had asthma at baseline. Age, BMI, heavy periods, or the presence of type 2 diabetes, hypertension, or depression at baseline did not affect the likelihood of iron deficiency at survey five.

Logistic regression analyses for baseline haem iron intake (2003) as a predictor of iron deficiency at survey 4 (2006) and survey 5 (2009) are presented in Table 3. The unadjusted analysis showed no statistically significant association between baseline haem iron intake and iron deficiency at either survey 4 or survey 5. After adjustment for potential confounders, baseline haem iron intake was a statistically significant predictor of iron deficiency at both survey 4 and survey 5. Replacing haem iron with energy-adjusted haem iron did not markedly change the associations at survey 4 or survey 5.

## 4. Discussion

This study estimated and described haem iron intake for a sample of young adult Australian women to determine the association between haem iron intake and the development of self-reported diagnosed iron deficiency over a six year period from 2003–2009. In adjusted analyses, baseline haem iron intake was shown to be associated with the risk of iron deficiency three and six years later. Cross-sectional analysis of an association between haem iron intake and iron deficiency reported at baseline was not conducted due to the possibility that iron deficiency (within the previous 3 years) occurred prior to the reported haem iron intake (within the previous 12 months). Covariate adjusted analysis revealed that a 1 mg/day increase in haem iron intake decreased the odds of developing iron deficiency by 10% three years from baseline and by 13% six years from baseline. The consistent association between baseline haem iron intake and iron deficiency over the two follow-up surveys, suggests that haem iron intake may be stable over time.

This is the first population based study to examine the association exclusively between haem iron intake and iron deficiency in young adult Australian women. Haem iron data is not readily available in most nutrient databases internationally and not at all in Australia. Therefore it was necessary to calculate haem iron values for certain Australian red meats, fish, and poultry to enable dietary analysis. It is not uncommon for haem iron databases to be created for research purposes. Sinha et al designed a FFQ and accompanying nutrient database to investigate how cooking times and temperatures affected certain compounds in meat, including haem iron [29]. Cross et al. (2012) recognised the lack of data for haem iron contents of foods for population based research and created a haem specific database for US foods in order to examine the relationship with disease outcomes [30]. Incorporating haem iron into nutrient databases for use with dietary assessment tools will help facilitate future research into the role of haem iron as a dietary predictor of iron deficiency, as well as enable more detailed examination of associations with any adverse health outcomes. However, it is clear when comparing the haem data from Cross et al. with Rangan et al., that haem databases are not readily transferrable across countries. Consistently, Rangan et al. reported higher haem contents for similar meat products cooked in similar ways, and the differences appeared to be the greatest for red meats (beef and lamb) [17]. As noted by Han et al., incomplete bleeding after slaughter, lower fat content, different breeds and feeding regimens, animal maturity, and sex could contribute to differences in meat iron content [31]. Livestock production in Australia differs considerably to other regions of the world, including the US. Most beef and lamb produced in Australia is fully grass fed, with only some very limited grain finishing before slaughter which is likely to result in leaner meat. Considerable work has also been done in Australia by pork producers to produce very lean pork meat, and the leanness of the meat is a feasible explanation for the higher haem contents, as fat marbling cannot be excluded through dissection like depot fat, and therefore ends up as part of the assay, diluting the haem content. Rangan et al. also reported a considerable difference in the haem content of Australian chicken which contained approximately 60% haem iron while chicken from other countries had haem iron content in the range of 20–40% of the total iron. This highlights the importance of country specific data on haem contents for epidemiological investigations on the role of haem in health and disease.

While this is one of the first population based studies to examine directly the association between haem iron intake and iron deficiency, our group has recently published a systematic literature review supporting a role for higher flesh food consumption in the prevention of iron deficiency [32]. This included all studies that had reported consumption of red meat, poultry, and fish, and any measure of iron status, including blood indices or self-report of diagnosed iron deficiency. Eight experimental studies and 41 observational studies were included, and while the results were somewhat inconsistent, especially the cross-sectional results, the high quality studies and interventions generally showed a positive association between flesh food consumption and iron status [32]. We concluded that the evidence for a relationship is promising, but that longitudinal analyses and more intervention studies are needed, especially to identify optimal intakes for the prevention of iron deficiency. The development of haem databases will significantly contribute to future research in this area.

Haem iron is a component of haemoglobin in red meat (beef, lamb, pork, and veal) and myoglobin in white meat (poultry and fish). The concentration of haem iron is considerably greater in haemoglobin (65%) compared with myoglobin (15%) [5]. As such, smaller servings of red meat relative to white meat provide equivalent amounts of haem iron. The quantities of flesh foods and mixed food items from the DQESv2 needed to supply an extra 1 mg dietary haem iron daily include: 59 g beef; 71 g lamb; 77 g veal; 111 g pork, ham or sausages; 143 g chicken, bacon or salami; 250 g fried fish; 333 g grilled or tinned fish; 111 g hamburger; 333 g pizza or 500 g pies. In terms of availability, portion size, and current consumption trends of the study sample, various cuts of fresh beef or lamb seem most appropriate for increasing haem iron intake. Ham, sausages, bacon, salami, pies, and certain pizzas contain high amounts of saturated fat and salt, thus it would not be desirable to increase consumption of these foods to provide extra haem iron daily [33]. 

There is the suggestion that haem iron may play a role in the development of certain chronic diseases due to its oxidative properties, inducing oxidative stress in the body and triggering inflammatory pathways and insulin resistance [34,35,36]. The literature focuses strongly on the potential link between dietary haem iron and colorectal cancer. A meta-analysis investigating haem iron from meat and the colorectal cancer risk from prospective cohort studies concluded there was a statistically significant association between haem iron and the development of colorectal cancer [35]. However, other compounds associated with meat intake such as saturated fats, nitrites in processed meats, as well as cooking methods which produce heterocyclic amines and polycyclic aromatic hydrocarbons could confound the association with cancer risk [33]. Two subsequent prospective studies have failed to show a statistically significant association between haem iron intake and increased cancer risk. Zhang et al. examined haem iron intakes and colorectal cancer risk in a study of men (40–75 years) and women (30–55 years). There was no strong evidence to support the hypothesis that haem iron intake increased colorectal cancer risk in either men or women [37]. Secondly, Kabat et al. measured haem iron intakes in post-menopausal women and showed that higher intakes of dietary haem iron were not significantly associated with an increased risk of breast cancer [36]. The development of tools to measure haem intakes among populations will allow further research in this area.

In Australia, the National Health and Medical Research Council (NHMRC) has developed evidence-based Australian Dietary Guidelines to assist the population in making healthy food choices for optimal wellbeing and the prevention of chronic disease [38]. The minimum recommended number of daily servings of lean meats, poultry, fish, eggs, nuts, seeds, legumes, and beans for women aged 19–50 years is 2.5 servings [33]. This provides an intake range between 163 g and 250 g of flesh food when the serving sizes for each of the different food items in the group is considered. However, it has been shown that the diets of Australian women do not align with these recommendations [39]. In the current study, the median daily intake of flesh foods and mixed food items including meat collectively was 124 g. The low consumption of meat and meat products by the study sample is perhaps attributable to demographics, as young adult women are typically health conscious, and the perceived desirability of flesh foods in Australia is probably affected by public health messages that advocate limiting the consumption of saturated fats [33]. The true health consequences of increasing haem iron intake remain inconclusive. However, the combined median intake of fresh red meat in this cohort was only 39 g/day (28 g/day beef + 9 g/day lamb + 2 g/day pork) and this level of intake is far below the upper limit of red meat consumption recommended by the NHMRC in the Australian Dietary Guidelines of 455 g/week or 65 g/day, suggesting that there is considerable scope to increase red meat consumption and therefore haem iron intake for those most at risk of iron deficiency, without exceeding recommendations [33]. Further research is needed to clarify the haem iron intakes of those groups at highest risk of iron deficiency, and to determine the optimal intake of flesh foods required to supply sufficient haem iron to avoid iron deficiency while minimising the risk of chronic disease.

A limitation of this study was the use of subjective self-reported data for analyses. Survey questions can be misinterpreted, resulting in misreporting. In addition, the survey questions relating to both dietary intakes and iron status required participants to report retrospectively. Consequently, recall bias is possible. In this study, it has necessarily been assumed that the participants’ dietary intakes remained constant from 2003 to 2009. We recognise that there is likely to have been dietary changes among the women, but we believe that significant findings of a relationship between self-reported iron deficiency and haem iron intakes three and six years earlier is even more convincing evidence of a role for haem iron. The consistency of the relationship between haem iron intake at baseline and iron deficiency 3 or 6 years later also suggests that at least over this time period, the intakes were stable. High heat and increased length of cooking time may reduce the haem iron contents of meat [29] but no information on this was available in the FFQ data. Other data that may have been important but was unavailable was iron supplement use. In later ALSWH surveys, a question about general vitamin and mineral supplement use was added, but information on iron supplements specifically is not available for any surveys.

A major strength of this study was the objective method of covariate selection [26,27]. The literature on iron deficiency suggests that the relationship between dietary iron intakes and iron status is weak due to confounding from many dietary, health, and lifestyle variables, such as vitamin C and fibre intakes, physical activity, chronic disease, blood donation, and in women specifically, menstrual blood loss and contraceptive use [1,12,15,40]. The DAG method allows the available data to identify covariates directly confounding the relationship between the independent and outcome variables, in this case the haem iron intake and iron deficiency, respectively, without researcher biases being introduced. While the data on menstrual blood loss in the ALSWH is very limited, neither it nor contraceptive use were found to confound the relationship between haem intake and iron deficiency. This is not to say that either of these variables is not related to iron deficiency among the ALSWH cohort, only that they are not relevant to the relationship with haem intakes and iron deficiency. The only dietary confounder identified by the DAG was alcohol consumption, which is a known enhancer of iron absorption but not generally thought to enhance haem intake. There is also non-haem iron in flesh foods, which may be influencing the relationship. Body Mass Index is also now considered to be an important determinant of iron absorption due to the effects of inflammation on hepcidin levels [41], but our data did not suggest any association between BMI and iron deficiency. In conclusion, the results of this longitudinal analysis support the hypothesis that higher haem iron intake is associated with reduced odds of iron deficiency developing in young adult Australian women. 

## Figures and Tables

**Table 1:** Iron present in the Dietary Questionnaire for Epidemiological Studies V2 (DQESv2) Food Frequency Questionnaire (FFQ) flesh foods and mixed foods and the intakes of these food items by Australian Longitudinal Study of Women’s Health (ALSWH) young cohort women in the study sample at survey three (baseline).

Flesh Food or Mixed Food (FFQ)	Total Iron (mg/100 g)	Haem Iron (mg/100 g)	Median Food Item Intake at Survey Three (g/Day)	Median Haem Iron Intake at Survey Three (mg/Day)
Beef	2.8	1.7	28	0.48
Lamb	2.4	1.4	9	0.13
Veal	2.1	1.3	0	0
Pork	1.4	0.9	2	0.02
				
Ham	1.4	0.9	3	0.03
Sausages/Frankfurters	2.5	0.9	3	0.03
Bacon	1.1	0.7	3	0.02
Salami	2.0	0.7	1	0.01
Chicken	1.2	0.7	26	0.18
Fish; fried	0.7	0.4	3	0.01
Fish; steamed, grilled, or baked	0.4	0.3	9	0.03
Fish; canned	1.4	0.3	5	0.02
Hamburger *	2.5	0.9	4	0.04
Pizza †	1.3	0.3	16	0.05
Pies ‡	1.2	0.2	12	0.02

Total Iron values from the DQESv2 database. Haem iron values generated from Rangan et al.’s experimentally determined haem contents for Australian meat and fish [17]. Food types from Rangan et al. that were used or averaged to determine haem contents (%) of DQESv2 flesh food items were: mince, rump steak, skirt steak, and rib roast for Beef and Veal (62%); chicken breast and thigh for Chicken (62%); lamb chop and lamb leg for Lamb (60.5%); pork chop for Pork (66%); ham for Ham (61%); bacon for Bacon (67%): beef sausage for Sausages or Frankfurters and Salami (36%); Snapper for fried, steamed/grilled/baked Fish (63%); Tuna for Canned fish (18%). For mixed food items, the assumptions and calculations follow: * Hamburger calculations: DQESv2 weight is 190 g, assume 105 g meat patty from the average of burger company websites (iron content from NUTTAB 3.99 mg), 60 g white roll (iron content 0.78 mg), thus iron for the total burger is 4.8 mg (2.5 mg/100 g) and iron from the meat patty is 3.99/4.8 = 83%. Rangan et al. [17] stated that the average of beef mince and sausage equals 42% haem, so the haem iron content of hamburger is (2.5 mg/100 g) × 0.83 × 0.42 = 0.86 mg haem iron/100 g. † Pizza calculations: DQESv2 weight is 278.8 g and total iron is 1.32 mg/100 g. Iron content Pizza base from NUTTAB is 1.3 mg/100 g. Assume the meat on pizza is 1.6 mg/100 g iron (average salami is 2.0 mg/100 g and ham is 1.4 mg/100 g) and assume the crust is 47% of the weight of the pizza (based on carbohydrate content of DQESv2 Pizza and NUTTAB pizza base 25/52.6); thus 1.3 × 0.47 = 0.61 mg iron from crust and 1.3 − 0.61 = 0.69 mg iron from the toppings (assumes all iron from toppings is from the meat, as the iron content of cheese and vegetables is minimal); Average haem iron content for salami (61%) and ham (36%) is 48.5%, thus the haem content of Pizza is 0.69 mg × 0.485 = 0.34 mg/100 g. ‡ Pie calculations: DQESv2 weight is 169.3 g and total iron is 1.21 mg/100 g. The Australian minimum standard for meat content is 25%, but the survey results show up to 38%, so assume 30% meat content. Using 48% haem content for minced beef [17], haem content pie is 1.21 mg × 0.30 × 0.48 = 0.17 mg/100 g.

**Table 2:** Characteristics of young cohort ALSWH women in the study sample at survey three (baseline) and associations with self-reported diagnosed iron deficiency at survey five.

Characteristic	No Iron Deficiency (*n* = 4599)	Diagnosis of Iron Deficiency (*n* = 766)	*p*-Value
Age in years	27.6 (1.45)	27.6 (1.45)	0.271
Mean (SD)			
Individual Income (%)			
<$37,000 annually	56.2	59.1	
>$37,000 annually	43.8	40.9	0.144
Highest education level (%)	33.6	29.9	
Year 10 or equivalent, trade/apprenticeship or diploma	17.5	17.8	
Year 12 or equivalent Bachelors or higher degree	49.0	52.3	0.124
Body Mass Index (kg/m^2^)	24.6 (5.29)	24.6 (5.58)	0.787
Mean (SD)			
Haem iron intake (mg/day)	1.38 (1.21)	1.26 (1.15)	0.0001
Median (IQR)			
Heavy menstruation (%)			
Sometimes	65.5	63.5	
Often	34.5	36.5	0.637
Alcohol (drinks/week)	0 (7)	0 (6)	0.0257
Median (IQR) *			
Smoking (cigarettes/week)	0 (0)	0 (0)	0.4754
Median (IQR) *			
Type 2 diabetes (%)	0.330	0.400	0.774
Asthma (%)	9.68	12.3	0.0260
Hypertension (%)	1.92	2.25	0.548
Depression (%)	11.2	12.5	0.319

SD, standard deviation; IQR, interquartile range. * Reported as whole units.

**Table 3:** Haem iron intake measured at survey 3 (baseline) and modelled as a predictor of self-reported diagnosed iron deficiency at survey 4 (2006) and survey 5 (2009) for Australian Longitudinal Study of Women’s Health young cohort women using univariate and multivariate logistic regression.

Predictor	Unadjusted Odds Ratio	Unadjusted 95% CI (*p*-Value)	Adjusted Odds Ratio *	Adjusted 95% CI (*p*-Value) *	Energy-Adjusted Odds Ratio †	Energy-Adjusted 95% CI (*p*-Value) †
Survey 4						
Haem Iron Intake (mg/day)	0.96	0.90, 1.02 (0.172)	0.91	0.84, 0.99 (0.020)	0.90	0.82, 1.00 (0.044)
Survey 5						
Haem Iron Intake (mg/day)	0.95	0.89, 1.01 (0.102)	0.89	0.82, 0.97 (0.007)	0.87	0.78, 0.96 (0.007)

* Adjusted for income, education, smoking, alcohol consumption. † Energy-adjusted haem iron intake using Willet’s residual method [28], adjusted for income, education, smoking, and alcohol consumption.
